# The Cluster [Re_6_Se_8_I_6_]^3−^ Induces Low Hemolysis of Human Erythrocytes* in Vitro*: Protective Effect of Albumin

**DOI:** 10.3390/ijms16011728

**Published:** 2015-01-13

**Authors:** Edgardo Rojas-Mancilla, Alexis Oyarce, Viviana Verdugo, Zhiping Zheng, Rodrigo Ramírez-Tagle

**Affiliations:** 1 Universidad Bernardo O’ Higgins, Departamento de Ciencias Químicas y Biológicas, General Gana 1780, Santiago 8370854, Chile; E-Mail: eseba21@gmail.com; 2 Universidad Bernardo O’Higgins, Escuela de Tecnología Médica, General Gana 1780, Santiago 8370854, Chile; E-Mails: alexis.oyarce@hotmail.com (A.O.); viviana.verdugo@live.com (V.V.); 3 Department of Chemistry, the University of Arizona, Tucson, AZ 85721, USA; E-Mail: zhiping@u.arizona.edu; 4 Universidad Bernardo O’ Higgins, Laboratorio de Bionanotecnología, General Gana 1780, Santiago 8370854, Chile

**Keywords:** cluster, hemolysis, erythrocytes, albumin

## Abstract

The cluster Re_6_Se_8_I_6_^3−^ has been shown to induce preferential cell death of a hepatic carcinoma cell line, thus becoming a promising anti-cancer drug. Whether this cluster induces acute hemolysis or if it interacts with albumin remains unclear. The effect of acute exposure of human red blood cells to different concentrations of the cluster with and without albumin is described. Red blood cells from healthy donors were isolated, diluted at 1% hematocrit and exposed to the cluster (25–150 µM) at 37 °C, under agitation. Hemolysis and morphology were analyzed at 1 and 24 h. The potential protection of 0.1% albumin was also evaluated. Exposition to therapeutic doses of the cluster did not induce acute hemolysis. Similar results were observed following 24 h of exposition, and albumin slightly reduced hemolysis levels. Furthermore, the cluster induced alteration in the morphology of red blood cells, and this was prevented by albumin. Together, these results indicate that the cluster Re_6_Se_8_I_6_^3−^ is not a hemolytic component and induces moderate morphological alterations of red blood cells at high doses, which are prevented by co-incubation with albumin. In conclusion, the cluster Re_6_Se_8_I_6_^3−^ could be intravenously administered in animals at therapeutic doses for* in vivo* studies.

## 1. Introduction

Success in cancer chemotherapy is based on the selectivity of some drugs to induce tumor cell death without affecting normal cells. In this context, some metal-based drugs appear to be promising in the development of efficient anticancer agents. Recent studies showed that the [Re_6_S_8_(OH)_6_]^4−^ hexarhenium hexahydroxo cluster and the [Re_6_S_8_(OH)_5_X]^4−^ X = OOC–LeuPheGlyLeuPheGly–NH–OCCH_2_(CH_2_CH_2_O)_12_OCH_3_ complex are internalized into cells and are mainly localized in the cytoplasm and nucleus. These clusters induce the suppression of cell proliferation and viability at concentrations higher than 100 µM with no cytotoxic effects at physiological concentrations (50 µM). Furthermore, the anionic hexa-iodo rhenium selenide cluster [Re_6_Se_8_I_6_]^3−^ induces 100% early tumor cell death, whereas the same concentration leaves normal cells unaffected, allows for the localization of tumors and enables the observation of tumor regression through fluorescence measurement during treatment. It is therefore concluded that the octahedral rhenium cluster complexes have promising potential for therapeutic and diagnostic applications [1,2,3,4].

Recent studies have shown that the K_4_[Re_6_S_8_(CN)_6_] cluster complex is mainly accumulated in the liver and can be excreted by the kidneys in rats. Moreover, it was also accumulated in the spleen in significant amounts [5].

Administration of drugs in live organisms implies transport through the cardiovascular system. The interaction of drugs with blood components could lead to diverse effects, including cell damage, formation of complexes with macromolecules and immune reactions [6]. The half-life of erythrocytes in circulation is about 110–120 days, when the spleen then eliminates them. Some diseases or exposure to certain substances, such as drugs, toxins or immunocomplexes, may cause premature breakdown of erythrocytes, thus impairing normal transport of oxygen and leading to hemolytic anemia. It is therefore relevant to establish whether a drug with therapeutic properties induces alterations in erythrocytes* in vitro* [7,8].

To establish whether cluster administration can induce hemolysis, human erythrocytes were exposed to different doses of the cluster and evaluated for possible hemolysis and morphological alterations [9]. In addition, the potential protective role of albumin was also evaluated.

## 2. Results and Discussion

**Figure 1:**
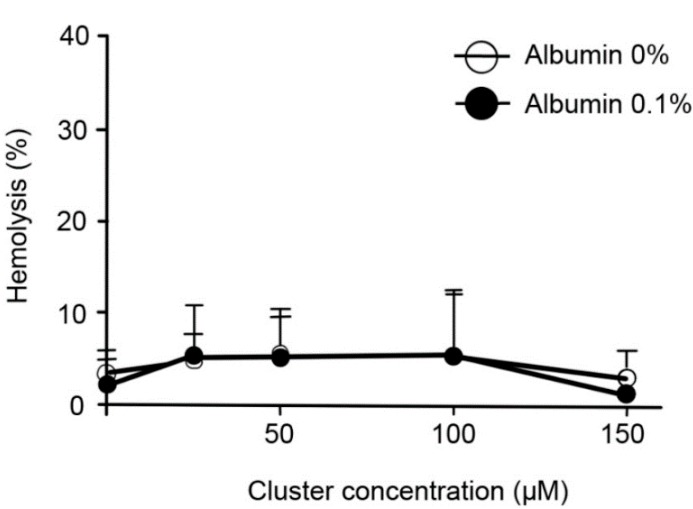
Acute exposition to increasing doses of the cluster did not induce hemolysis* in vitro*. The percentage of hemolysis after 1 h of incubation is shown. Erythrocytes suspended in distilled water were considered 100% hemolyzed. High doses of the cluster did not increase hemolysis* in vitro* in the presence or absence of albumin, compared to controls.

**Figure 2:**
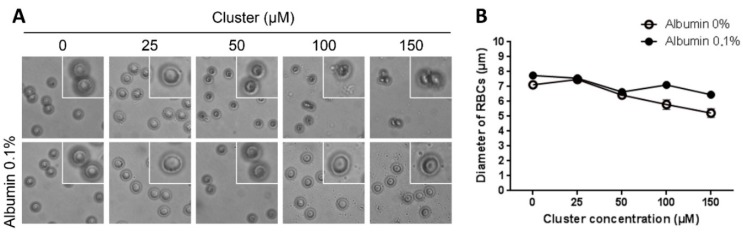
Morphology of human erythrocytes after acute exposition to different concentrations of the cluster with or without albumin. (**A**) Representative pictures of the experimental conditions are shown. Exposition to 100 and 150 µM of the cluster without albumin leads to crenation of erythrocytes, compared to controls; and (**B**) The size of erythrocytes is significantly reduced at high doses of the cluster (*p* < 0.05). Both morphological alterations and reduction in size were prevented under co-incubation with albumin.

**Figure 3:**
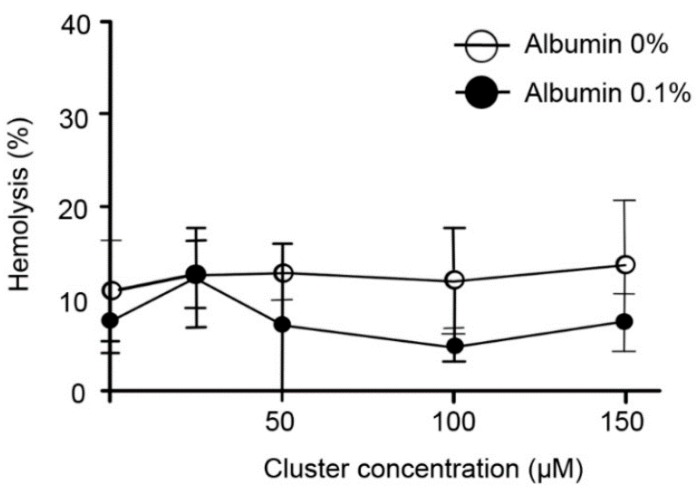
Exposition during 24 h to increasing doses of the cluster did not induce hemolysis* in vitro*. The percentage of hemolysis after 24 h of incubation is shown. Erythrocytes suspended in distilled water were considered 100% hemolyzed. High doses of the cluster did not increase hemolysis* in vitro*, compared to controls, and albumin co-incubation showed a tendency to reduce hemolysis (not significant).

**Figure 4:**
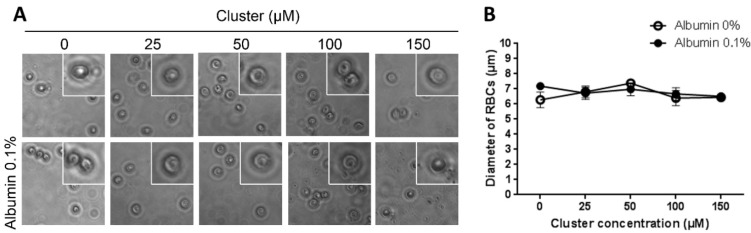
Morphology of human erythrocytes after 24 h of exposition to different concentrations of the cluster with or without albumin. (**A**) Representative pictures of the experimental conditions are shown; and (**B**) The average size of erythrocytes in all conditions is similar, but reduced compared to fresh erythrocytes. Albumin reached similar results.

Acute exposition of human erythrocytes to therapeutic doses of the cluster did not induce hemolysis* in vitro* (Figure 1). Additionally, acute exposition to high doses of the cluster induced alterations in the morphology of erythrocytes (Figure 2A); the normal size of erythrocytes ranged between 7 and 8 µm. Exposition to 100 and 150 µM of the cluster leads to a significant reduction in cell size (6.1 and 5.6 µm, respectively) compared to controls (7.3 µm, Figure 2B). Erythrocytes exposed to 100–150 µM of the cluster showed echinocytosis and crenation. Both morphological alterations and reduction in size were prevented by co-incubation with albumin (Figure 2). Similarly, very low hemolysis was observed after 24 h of incubation at 37 °C and under agitation (Figure 3). Furthermore, a reduction in the size of the erythrocytes was observed in all experimental conditions, with an average size of 6.5–7.0 µm (Figure 4).

Regarding the productivity of anticancer metallodrug discovery and development, there is limited knowledge about how or in which metabolic state the metal complex penetrates the tumor cell and how much of the complex is inactivated. In view of the fact that a vast majority of cytotoxic metal-containing compounds are administered intravenously, special considerations should therefore be given to the interactions of the metal drugs with the macromolecular and cellular components of the blood [10], which can then be taken up by and accumulated in tumor tissue. In this context, regarding binding to serum proteins that may perform a transport function for metallodrugs, albumin appears to be the most important issue [11,12], because such interactions also determine the overall drug distribution and excretion and the differences in efficacy, activity and toxicity [13,14].

Hemolysis is the breakage of the erythrocyte membrane, with the release of hemoglobin to the plasma, changing its color to pink or red. This reduces the capacity to transport oxygen, a condition named hemolytic anemia [15]. We examined the hemolytic properties of increasing doses of the cluster Re_6_Se_8_I_6_^3−^ in human erythrocytes* in vitro*, finding low hemolysis levels at therapeutic concentrations.

It has been reported that TiO_2_ nanoparticles induce hemolysis of human erythrocytes* in vitro*, characterized by spherocytosis and echinocytosis [16].

This* in vitro* study needs to be corroborated by* in vivo* analysis of hemolytic properties, thus ensuring that administration in animals does not induce hemolytic anemia [17].

## 3. Experimental Section

### 3.1. Synthesis of ([n-Bu_4_N]_3_[Re_6_Se_8_I_6_])^3−^

TBA (tetrabutyl ammonium) salt of the octahedral hexarhenium(III) (one of the 6 Re is actually Re(IV)) chalcogenide cluster complex, Re_6_Se_8_I_6_^3^^−^ cluster, was prepared and purified according to procedures reported elsewhere [1,18,19]. A stock solution of the cluster in DMSO at 100 mM was prepared and used within the day. Tetrabutylammonium iodide solution was used as a control (Figure 1).

### 3.2. Dose Selection

Erythrocytes were exposed to increasing concentrations of the cluster (25 to 150 µM). These concentrations were selected from previous studies reporting biological activity of the cluster at 50 µM in the HepG2 cell line [1].

### 3.3. Human Blood Samples and Preparation of Erythrocytes

**Figure 5:**
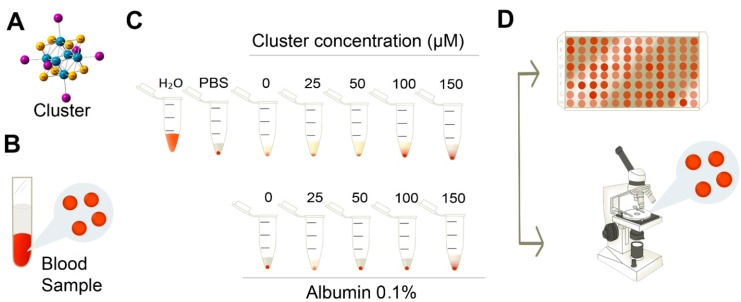
Scheme of experimental procedure. (**A**) Hexanuclear rhenium [Re_6_Se_8_I_6_]^3−^ cluster complex; (**B**) to test the hemolysis effects of the cluster on erythrocytes* in vitro*, blood samples from human donors were obtained, and erythrocytes were isolated; (**C**) erythrocytes (1%) were exposed to different concentrations of the cluster. Suspension in distilled water was considered the control of 100% hemolysis; (**D**) Absorbance of free hemoglobin was measured in an ELISA plate, to estimate % of hemolysis. The morphology of erythrocytes was analyzed under an inverted microscope.

Blood samples were obtained from human voluntary donors. Donors agreed to the use of their blood in a hemolysis study by signing an informed consent form. The local bioethics committee approved the study. Blood samples were obtained through venipuncture (2.7 mL), anticoagulated in a tube with sodium citrate 3.2% (BD Vacutainer, Franklin Lakes, NJ, USA) and gently homogenized. Erythrocytes were isolated from blood components by centrifugation (Eppendorf 5804R, Hamburg, Germany) at 3500 rpm during 5 min at room temperature. Erythrocytes were immediately transferred to 15-mL falcon tubes and washed three times with phosphate-buffered saline (PBS; NaCl 150 mM, NaH_2_PO_4_ 1.9 mM, Na_2_HPO_4_ 8.1 mM, pH 7.4). Concentrated erythrocytes (hematocrit, 50%) were used immediately after isolation (Figure 5).

### 3.4. Test of Hemolysis

Erythrocytes (hematocrit, 1%) were treated with different cluster concentrations (25, 50, 100, 150 µM). In parallel, a similar set of concentrations was supplemented with 0.1% albumin. A suspension of erythrocytes treated with tetrabutylammonium was included. Erythrocytes suspended in distilled water or PBS were considered as controls of 100% or 0% of hemolysis, respectively (Figure 5).

Tubes were placed in an incubator (Binder BD115, Tuttlingen, Germany) at 37 °C and agitated by an orbital shaker (Vision Scientific VS-201D, Geyonggi-do, Korea) at 70 rpm for 1 h. Then, tubes were gently homogenized, and a direct sample was prepared (30 µL) on a slide and covered with a coverslip. This was maintained in a humid chamber, pending observation under a microscope. An aliquot (100 µL) was centrifuged at 3500 rpm for 5 min at room temperature. The supernatant was transferred to a 96-well plate (Falcon, Tewksbury, MA, USA) for hemoglobin determination. The absorbance of supernatants was read at 540 nm in an ELISA reader (Tecan, Infinite, Grodig, Austria) (Figure 1).

### 3.5. Morphology of Erythrocytes

Direct samples were examined in an optical inverted microscope (MOTIC instruments, AE31, Richmond, Canada). Pictures of representative areas at 40× were obtained. The morphology of erythrocytes was analyzed with ImageJ software (NIH, Bethesda, MD, USA) (Figure 1).

### 3.6. Data Analysis

All results are presented as the mean ± SD. The Student’s *t*-test and ANOVA, followed by the Bonferroni or Dunn’s *post hoc* tests, were used and considered significant at *p* < 0.05.

## 4. Conclusions

We observed that treatment with therapeutic or high doses of the cluster induced minimal hemolysis* in vitro*. Similar results were observed following 24 h of exposition. Furthermore, higher doses of the cluster altered red blood cell morphology, and albumin prevented this effect. Together, these results indicate that the cluster Re_6_Se_8_I_6_^3−^ induces minimal hemolysis and morphological alterations of red blood cells at therapeutic doses, and morphological alterations were prevented by albumin. The cluster Re_6_Se_8_I_6_^3−^ could be administered to animals at therapeutic doses for* in vivo* studies. Considering that alterations in erythrocytes could lead to hemolysis, leading to hemolytic anemia, safe administration* in vivo* is a fundamental issue that needs to be evaluated.
